# Exploring Direct 3D Interaction for Full Horizontal Parallax Light Field Displays Using Leap Motion Controller

**DOI:** 10.3390/s150408642

**Published:** 2015-04-14

**Authors:** Vamsi Kiran Adhikarla, Jaka Sodnik, Peter Szolgay, Grega Jakus

**Affiliations:** 1 Holografika, Baross u. 3. H-1192 Budapest, Hungary; 2 Faculty of Information Technology, Pazmany Peter Catholic University, Prater u. 50/a, Budapest 1083, Hungary; E-Mail: szolgay.peter@itk.ppke.hu; 3 Faculty of Electrical Engineering, University of Ljubljana, Tržaška 25, Ljubljana 1000, Slovenia; E-Mails: jaka.sodnik@fe.uni-lj.si (J.S.); grega.jakus@fe.uni-lj.si (G.J.)

**Keywords:** 3D display, light field display, free-hand interaction, direct touch, Leap Motion Controller, human-machine interface (HMI), human-computer interface (HCI)

## Abstract

This paper reports on the design and evaluation of direct 3D gesture interaction with a full horizontal parallax light field display. A light field display defines a visual scene using directional light beams emitted from multiple light sources as if they are emitted from scene points. Each scene point is rendered individually resulting in more realistic and accurate 3D visualization compared to other 3D displaying technologies. We propose an interaction setup combining the visualization of objects within the Field Of View (FOV) of a light field display and their selection through freehand gesture tracked by the Leap Motion Controller. The accuracy and usefulness of the proposed interaction setup was also evaluated in a user study with test subjects. The results of the study revealed high user preference for free hand interaction with light field display as well as relatively low cognitive demand of this technique. Further, our results also revealed some limitations and adjustments of the proposed setup to be addressed in future work.

## 1. Introduction

Light field display is a major breakthrough among glasses free 3D displaying technologies and provides very natural representation of 3D scenes. The underlying principle of displaying 3D content is explained by legacy Stereoscopic 3D (S3D) technology. 3D illusion in S3D is created by providing two slightly offset views of a scene captured by two cameras simultaneously and synchronized to left and right eyes of the viewer. View isolation is achieved by using special eye-wear.

In a glasses free system, the process of view isolation has to be part of display hardware and such displays are, therefore, generally called *autostereoscopic displays*. To achieve the separation of views, the intensity and color of emitted light from every single pixel on the display should be a function of direction. The adjustment of directionally dependent light beams for providing a displacement in the apparent position of an object viewed along two different line of sights (*i.e*., the parallax) will lead to more realistic 3D visualization compared to S3D. In autostereoscopic displays, the transmission of light is directed by employing parallax barriers and lenticular lenses. The effective 3D Field Of View (FOV) and angular resolution are functions of number of barriers. Due to the practical limitations of the hardware the final FOV of multiview autostereoscopic displays is rather limited.

**Figure 1:**
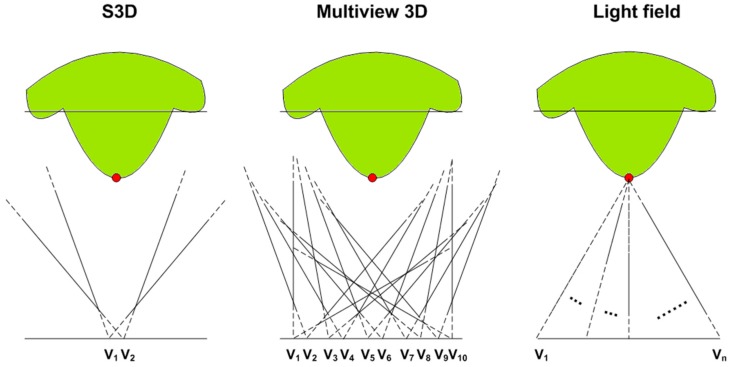
Displaying in 3D using Stereoscopic 3D (S3D), multiview 3D and light field technologies.

State-of-the-art wide FOV light field displays use a holographic diffuser instead for directive light transmission. Although alternative methods based on time multiplexing for establishing the light field exist (e.g., [1]), they require very high refresh rates to comply with the angular resolution and FOV of hologram-based light field displays. Such high refresh rates are hard to achieve with existing consumer devices and the spatial details become less perceivable. In case of holographic light field displays, the directional light emitted from all the points on the screen creates a dense light field, which, on one hand, creates stereoscopic depth illusion and on the other hand, produces the desirable motion parallax without involving any multiplexing. Figure 1 gives an overview of traditional S3D, multiview 3D and light field displaying technologies. 

As shown in Figure 1, consider a sample scene (shown in green) and a point in the scene (shown in red). From the rendering aspect, the major difference is that S3D and multiview rendering do not consider the positions of 3D scene points. Therefore we have only two perspectives of a given scene on a S3D display and multiple but a still limited number of perspectives on a multiview 3D display. In both the cases, all perspectives are actually 2D projections of the 3D image, which collectively define the scene. Light field displays in contrast define the scene using directional light beams emitted from the scene points. Thus each scene point is rendered differently from other scene points resulting in more realistic and accurate 3D visualization.

**Figure 2:**
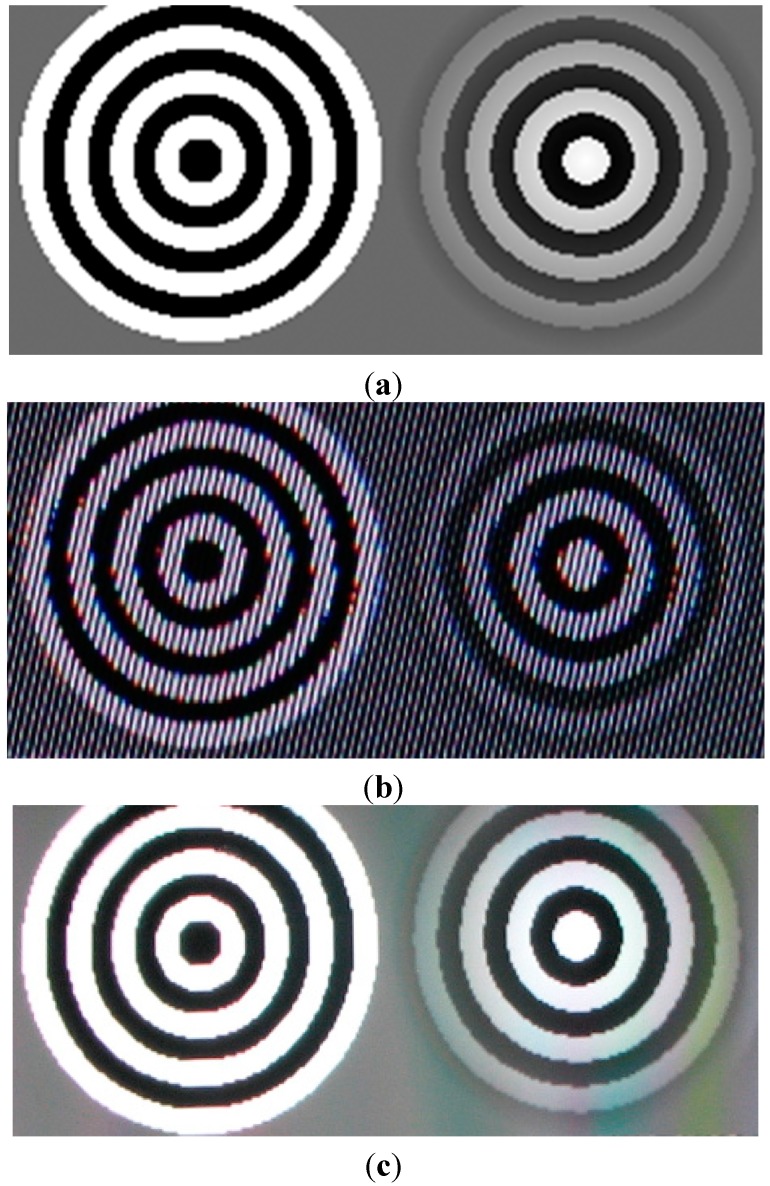
Light field and multiview autostereoscopic display comparison (**a**) Original 2D input patterns; (**b**) Screen shot of multiview autostereoscopic display; (**c**) Screen shot of projection-based light field display.

A direct advantage of light field displays can be clearly observed from Figure 2. Figure 2a shows two patterns of concentric circles lying in a plane. Figure 2b shows the screen shot of the patterns visualized on a barrier based multiview autostereoscopic display while Figure 2c shows the screen shot of a light field display.

As the number of views and effective FOV in horizontal direction are different for two displays, for a fair comparison all the views are engaged with the same 2D pattern when recording the screen shots. In case of multiview autostereoscopic displays, we have a limited number of comfortable 3D viewing zones called sweet spots. Within these zones a user can see an accurate 3D image while within the transitions between two neighboring sweet spots the image is blurry and disturbing. A user located anywhere within the FOV of multiview displays can always see mesh-like parallax barrier (Figure 2b). The size of the barrier is a major limitation in the display hardware and the perspective shift for motion parallax is neither smooth nor uniform. Another inherent drawback of the parallax barrier based approach is the limitation of the total achievable 3D FOV. In case of light field displays, there are no sweet spots and no light barriers. The diffusing properties of the screen provide continuous-like motion parallax and support wide FOV [2].

As light field displays represent a novel technology in the field of 3D rendering, they also require design and evaluation of novel interaction technologies and techniques for successful manipulation of displayed content. In contrast to classic interaction with 2D content, where mice, keyboards or other specialized input devices (e.g., joystick, touch pad, voice commands) are used, no such generic devices, techniques, and metaphors have been proposed for interaction with 3D content [3]. The selected interaction techniques usually strongly depend on individual application requirements, design of tasks and also individual user and contextual properties. Majority of 3D applications, especially those including virtual worlds and environments, seek for more natural and intuitive interaction resulting in usable and motivating application [4]. For this purpose, the use of special sensors is often required enabling real-time tracking of selected body parts and corresponding gestures, potentially with multiple degrees-of-freedom and minimum obstructive interference.

In this paper, we do not focus on a specific type of interaction with light field displays but rather propose a general solution for integration of a motion tracking sensor as input device and a light field display as output device. The main goal is to enable accurate, natural and intuitive freehand interaction with 3D objects rendered on a light field display. For this purpose a basic and most intuitive interaction method in 3D space, known as “direct touch” is applied. The direct touch can, for example, be used for selection of items by directly pointing and touching their virtual representations in the scene. Such method directly links an input device with a display and integrates both into a single interface [5]. The main challenge of the proposed setup is the selection and implementation of the most suitable input device allowing efficient interaction and accurate alignment and calibration with the output rendering.

The direct touch interaction requires an input device capable of precise tracking of human hands and fingers. In our study, we used the Leap Motion Controller [6], which is one of the most recently developed devices for accurate tracking of these body parts with the performance that is expected to enable fully exploiting the advantages of light field displays.

The most important contributions of this paper are the following:

- We propose the first framework that provides a realistic direct haptic interaction with virtual 3D objects rendered on a light field display. Our solution consists of a calibration procedure that leverages the available depth of field and the finger tracking accuracy, and a real-time interactive rendering pipeline that modifies and renders light field according to 3D light field geometry and the input gestures captured by the Leap Motion Controller.
- We evaluate the implemented interaction framework and report on the results of a first user study on interaction with a light field display. The aim of the study was a subjective and objective evaluation of the proposed interaction setup and user experience when interacting with the content rendered on light field displays.

To our knowledge this is the first research involving free hand interaction with light field displays. 

The remainder of this paper is organized as follows. The following section presents the related work on the interaction with 3D content. In Section 3, we describe the integration of the input device with the light field display and the calibration method, which enables direct touch interaction with the content rendered in 3D. Section 4 presents the evaluation design and the results of the evaluation. The paper concludes with the discussion and future work in this field.

## 2. Related Work

The devices that enable the interaction with 3D content are generally categorized into two groups, which correspond to wearable and hands-free input devices. The devices from the first group need to be physically worn or held in hands, while, on the other hand, no physical contact between the equipment and the user is needed when using hands-free devices.

### 2.1. Wearable Devices

One of the recent commercially successful representatives of wearable devices was the Nintendo WiiMote controller serving as the input device to the Wii console, released in 2006. The device enables multimodal interaction through vocal and haptic channels but it also enables gesture tracking. The device was used for 3D interaction in many cases (see for example [7]), especially to track the orientation of individual body parts. On the other hand, it is less appropriate for precise object manipulation due to its lower accuracy and relatively large physical dimensions.

### 2.2. Marker-Based Optical Tracking Systems

As wearable devices in general (including data gloves) impede the use of hands when performing real world activities, hand movement may also be tracked visually using special markers attached to the tracked body parts. Optical tracking systems, for example, operate by emitting infra-red (IR) light to the calibrated space. The IR light is then reflected from highly-reflective markers back to the cameras. The captured images are used to compute the locations of the individual markers in order to determine the position and orientation of tracked body parts. The advantage of the approach is a relatively large interaction volume covered by the system; while the disadvantage is represented by the fact that the user still has to wear markers in order to be tracked.

The optical tracking system was, for example, used as the tracking device when touching the objects rendered with a stereoscopic display [8]. The results of the study demonstrated the 2D touch technique as more efficient when touching objects close to the display, whereas for targets further away from the display, 3D selection was more efficient.

Another study on the interaction with a 3D display is presented in [9]. The authors used optical tracking system to track the positions of markers placed on the user’s fingers for a direct gestural interaction with the virtual objects, displayed through a hemispherical 3D display.

### 2.3. Hands-Free Tracking

Optical tracking can also be used for marker-less hands-free tracking. In this case, the light is reflected back from the body surface and the users do not need to wear markers. However, as body surface reflects less light compared to highly-reflective markers, this usually results in a much smaller interaction volume.

Although a number of studies with hands-free tracking for 3D interaction have been performed with various input setups (e.g., [10,11]), Microsoft Kinect sensor represents an important milestone in commercially accessible hands-free tracking devices. The device was introduced in late 2012 as an add-on for the Xbox 360 console. Beside visual and auditory inputs, the Kinect includes a depth-sensing camera, which can be used to acquire and recognize body gestures for multiple users simultaneously [12]. The device proved to be mostly appropriate for tracking whole body parts (*i.e*., skeletal tracking), e.g., arms and legs while it is less appropriate for finger and hand tracking.

A variety of studies using Kinect and other camera-based approaches have been conducted including studies on the interaction with a 3D display (e.g., [11,13,14,15]). A study similar to the one presented in this paper was conducted by Chan *et al*. [5] where users had to perform selecting tasks by touching the images rendered on an intangible display. The touch detection was implemented using stereovision technique with two IR cameras. The display used in the study was based on projection of a flat LCD screen to a fixed virtual plane in front of the user. Consequentially, only 2D planar images were displayed, resulting in limited range of viewing angles and no true depth perception as it is provided by volumetric displays.

### 2.4. Leap Motion Controller

Another important contribution to the affordable desktop hands-free motion sensing devices was the release of Leap Motion Controller [6] in 2013. The device, approximately the size of a matchbox, is placed on the desktop in front of the computer screen. It enables continuous tracking of multiple hands with up to a fraction of a millimeter accuracy [16], which allows the interaction with individual 3D voxels of a light field display.

A variety of studies involving Leap Motion Controller have been performed including studies with 3D gestures [17,18,19] and pointing tasks [20,21]. These approaches are based on defining a set of gestures for interaction often involving feedback to the user in the form of virtual pointers in 3D space, which are rendered inside the scene. These pointers guide a user by visually showing the active hand/finger position and the scene for interaction is presented on a normal 2D display.

### 2.5. Freehand Interaction with Projection-Based Light Field Displays

Approaches presented in [22,23] propose a gesture-based interaction framework for light field displays using Leap Motion Controller. The proposed approaches enable the manipulation of three-dimensional objects with seven degrees of freedom and leverage natural and familiar gestures. However, the proposed approaches and setups are not sufficiently evaluated objectively or subjectively. Moreover, interaction and display spaces remain isolated and these approaches do not explicitly take into account the display characteristics in terms of both geometry and resolution of the reproduced light fields for interaction.

In the current work, we propose a framework to explore direct interaction with virtual objects on a light field display. We couple the interaction and display spaces to provide an illusion of touching virtual objects. To the best of our knowledge, we report on the first study involving direct interaction with virtual objects on a light field display using Leap Motion Controller. The proposed interaction setup is very general and is applicable to any 3D display without glasses. In the current work we explore our 3D interaction framework for holographic light field display with full horizontal parallax use. However, the method is easily extendable to 3D displays with vertical parallax as well. In addition, our method is scalable, and the interaction space can easily be extended by integrating multiple Leap Motion Controllers.

## 3. System Design

### 3.1. Leap Motion Controller

**Figure 3:**
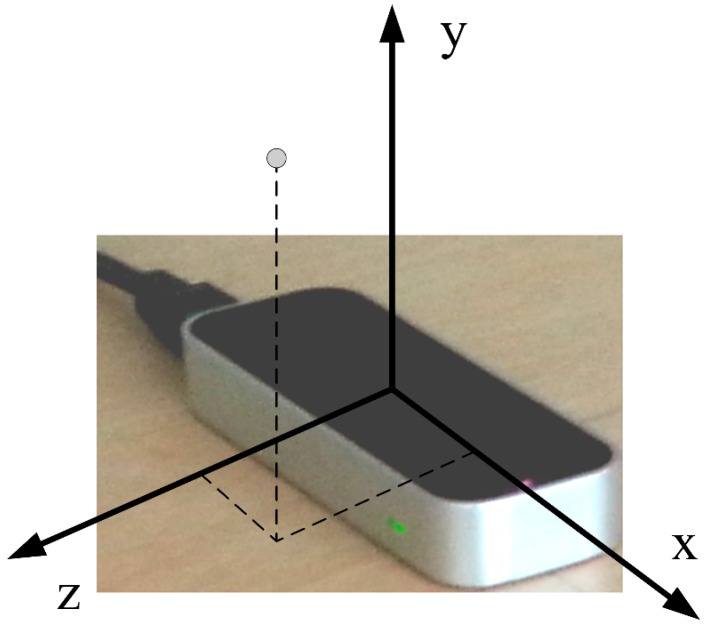
Leap Motion Controller and the coordinate system used to describe positions in its sensory space.

The Leap Motion Controller can be categorized into optical tracking systems based on stereovision. The device uses three LED emitters to illuminate the surrounding space with IR light, which is reflected back from the nearby objects and captured by two IR cameras. The device’s software analyzes the captured images in real-time, determines the positions of objects and performs the recognition of user’s hands and fingers. The discrete positions of recognized hands, fingers and other objects as well as detected gestures can then be obtained through APIs (Application Programming Interfaces). The device and the coordinate system used to describe positions in the device’s sensory space are shown in Figure 3.

A study on the Controller’s performance [16] revealed that the device’s FOV is an inverted pyramid centered on the device. The effective range of the Controller extends from approximately 3 to 30 cm above the device (*y*-axis), approximately 0 to 20 cm behind the device (negative *z*-axis) and 20 cm in each direction along the device (*x*-axis). Standard deviation of the measured position of a static object was shown to be less than 0.5 mm.

### 3.2. 3D Rendering on Light Field Displays

Light field displays are of high resolution (order of magnitude of one million pixels) and can be used by several users simultaneously. There is no head-tracking involved and thus the light field is available from all the perspectives at any given instance of time. For the experiment, we used a small-scale light field display of the size comparable to the FOV volume of Leap Motion Controller. The light field display hardware used for this work was developed by Holografika.

**Figure 4:**
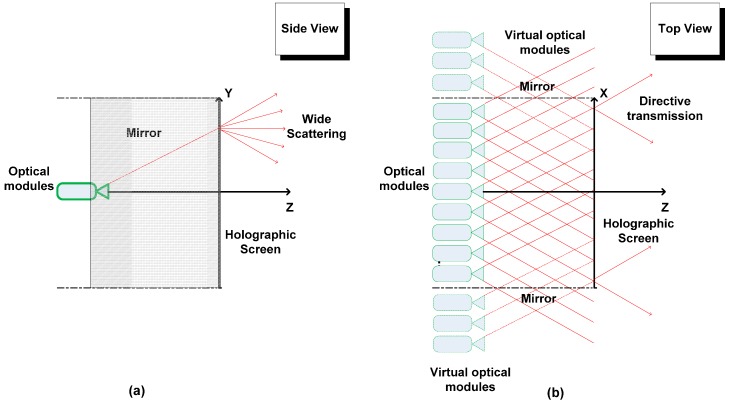
Main components of a light field display: geometrically aligned multiple optical modules, a holographic screen and side mirrors which help in increasing the field of view. (**a**) Horizontally holographic screen allows directive light transmission; (**b**) Vertically, the screen scatters light beams widely such that the projected image can be viewed from any height.

The display uses a specially arranged array of optical modules driven by a single computer, a holographic screen and two mirrors along the sidewalls of the display (see Figure 4). The screen is a flat hologram and the optical modules are arranged densely at a constant distance from the screen. The light beams emitted from the optical modules hit the holographic screen, which modulates them to create the so-called light field. Two light rays emitted from two optical modules crossing in space define a scene point. In real world the term light field is a function that describes the amount of light faring in every single direction through all the points in space. For realization light field is often defined over a single planar surface. In reality, the directions and light beams emitted from a point in space are continuous. In practice, however, it is not possible to imitate such continuousness due to the non-negligible size of the display hardware, which results in the discretization of the light beam directions.

The discretization of direction incorporated by light field displays leaves us a parameter to choose, the angular resolution. High angular resolution drives us closer to the real world at the cost of increased data to handle and *vice versa*. The angular resolution and the total FOV are directly proportional to the number of optical modules. The optical properties of the screen allow directional transmission of light in horizontal direction with minimum aliasing (see Figure 4b). If the input light beam is perfectly coherent, there will be no aliasing. In vertical direction, after hitting the screen the light beams scatter widely and thus users see exactly the same image at any height on the display. Such an arrangement can be used to create a horizontal-only parallax display (see Figure 4a). Mirrors covering the display sidewalls reflect back any light beams hitting them towards the screen, giving an illusion that they are emitted from a virtual light source outside the display walls (see Figure 4b). Thus the side mirrors increase the effective FOV by utilizing all the emitted light rays.

Light field displays require generating multiple light field slices along each available perspective. As in other state-of-the art rendering methods for such displays, we exploit multiple center of projection (MCOP) geometries [24] and adaptive sampling [25] to fit with the display geometry and the angular resolution of light beams. The method is based on the approach of fixing the viewer’s height and distance from the screen to those of a virtual observer for providing horizontal parallax. By appropriately modeling the display geometry, the light beams leaving the various pixels can be made to propagate in specific directions, as if they were emitted from physical objects at fixed spatial location. Users moving freely in front of the display can have an illusion of virtual objects floating in air. Note that the objects closer to the screen appear sharper than others as the spatial resolution of the display is depth dependent. To enable better viewing, the optical modules should be made to focus on a specific plane, which is usually the screen surface. Due to the incoherence of the emitted light, the beams tend to diverge, creating an aperture angle. When defining scene points at a given depth, the radius of these points formed by crossing light rays is a function of aperture angle. As beams diverge more and more with distance from screen, this results in a varying spatial resolution.

**Figure 5:**
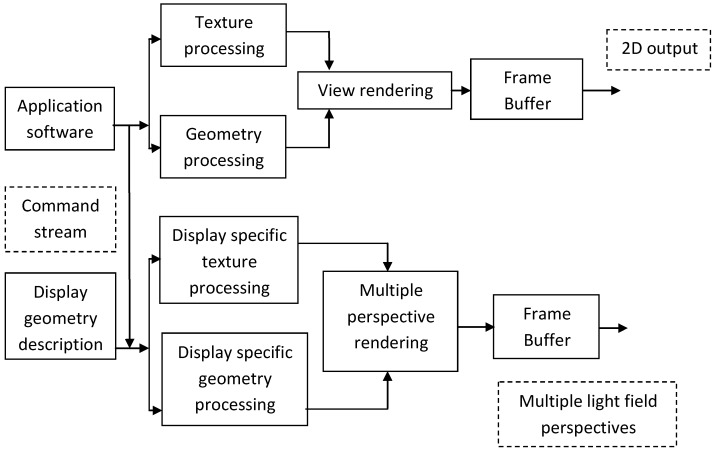
Light field rendering from OpenGL command stream: the various commands from application software are modified in real-time using the display geometry description. Geometry and texture information is modified and processed to render multi-perspective light field.

Real-time visualization on light field displays requires rendering the given scene from many viewpoints that correspond to the characteristics of the specific light field display. One way to achieve this is using the HoloVizio OpenGL wrapper [26]. This wrapper library intercepts all OpenGL calls and sends rendering commands over the network to the backend driving the light field display as well as modify related data (such as textures, vertex arrays, VBOs, shaders, *etc*.) on the fly to suit the specifications of the actual light field display. The wrapper functionality is shown in Figure 5. The wrapper is designed in such a way that its operation is completely transparent to the client application producing the scene and it requires no modification of the client application (in the case of third-party applications such modifications are usually not possible) [22].

For our registration procedure, we need to know the positions of real-world vertices in the screen coordinate space. For this purpose, we draw a scene of volume exactly the same as the displayable volume. When mapping the application’s Region of Interest (ROI) to the light field display’s ROI we add an additional constraint to map the central plane of the scene to the screen plane. This ensures correct mapping of scene coordinates to display coordinates. We customize OpenGL wrapper with this additional semantic information to match the two ROIs.

### 3.3. Experimental Setup

**Figure 6:**
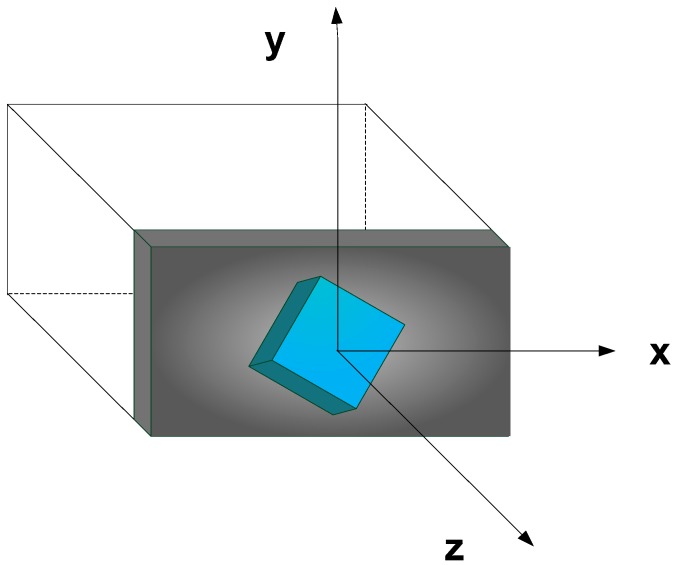
Display right hand coordinate system: screen lies along the plane *z* = 0, *x-*axis pointing to the right, *y* pointing to the vertical direction and *z-*axis out of the screen.

**Figure 7:**
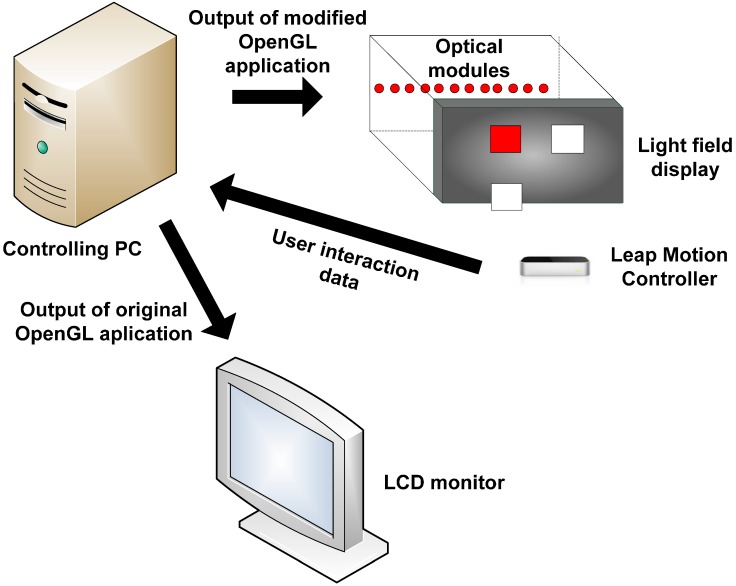
Experimental setup: The controlling PC runs two applications: main OpenGL frontend rendering application for 2D LCD display and backend wrapper application that tracks the commands in current instance of OpenGL (front end application) and generates modified stream for light field rendering. The front end rendering application also receives and processes user interaction commands from Leap Motion Controller in real-time.

We assume that the screen is located at *z =* 0 with center as the origin of the display coordinate system. The *y-*axis is in the vertical direction, the *x*-axis pointing to the right and the *z*-axis pointing out of the screen. The display coordinate system is shown in Figure 6 and experimental setup is shown in Figure 7. Leap Motion Controller is placed in front of the display with *x-, y-* and *z*-axis parallel to display *x-, y-* and *z*-axis. 

All the rendering and interaction functionality is implemented on single computer. The pattern for interaction is implemented in C++ using OpenGL. The application generates random patterns of tiles in run time and rendered at a given depth. In parallel, the application receives interaction data from Leap Motion Controller, processes and updates the renderer in real-time. The controlling PC runs GL wrapper and feeds the resulting visual data to optical modules. Hence we can see the same application running on a LCD monitor in 2D and on light field display in 3D.

### 3.4. Calibrating Light Field Display to Leap Motion Controller

For calibration, we assume that the display is at a fixed position with Leap Motion Controller placed anywhere in front of it. The exact position of the Controller in the display coordinates is not known. To ensure uniformity, we assume that both the display and Controller coordinates are in real world millimeters. In practice, when using Leap Motion Controller, hand-positioning data can be more accurately acquired at heights greater than 100 mm [16]. To meet this requirement, we place the Controller at a height less than h_max_, the maximum allowed height from the display center, where h_max_ is given by the Equation (1) and $D_{h}$ is the height of the screen in millimeters.

(1) hmax=(Dh2)−100mm

As it is not possible physically to reach the zones of the display where depth values (on the *z*-axis) are negative, we only consider the depth planes on and above the surface of the display for interaction. We follow an approach based on sparse markers for performing the calibration. A set of spherical markers centered at various known positions in display coordinates are rendered on the light field display and user has to point to the centers of the projected spheres one after another sequentially with index finger. The positions of the pointed centers as seen by the user (the fingertip positions) are recorded in Leap Motion Controller coordinate system. This information serves as an input for calculating the transfer function between the two coordinate systems. 

Given that the display projection modules are pre-aligned and calibrated (display geometry calibration independent of Leap Motion Controller), without the loss of generality, we can assume that both the display and Leap Motion Controller coordinates are Cartesian. Thus, in theory a rigid calibration should be possible, which means we only have a translation and rotation between the two 3D volumes and the distance between any two points would be same in both the spaces. However, in practice, this is not the case due to the characteristics of light field displays. As mentioned in Section 3.2, the spatial resolution of a light field display is not uniform all over the workspace and depends on depth. This means that the apparent size of the calibration marker will not be the same when projected on the surface of the screen and elsewhere. Also, the display geometry calibration data is calculated based on minimizing the projection error on the surface of the screen. Thus, similar to spatial resolution, the calibration accuracy will not be the same all over and is spatially varying. 

One of the outcomes of reduced calibration accuracy is blurring. Although a blurred background far from the user is acceptable, excessive blur near the user leads to discomfort. Also, Leap Motion Controller has relatively high frame rates and can track minor finger/hand movements. A minute finger shaking during the calibration can substantially reduce the calibration accuracy. Hence, given the size of the display and the precision of the Controller (up to 1/100 of an mm) we should take all the aforementioned effects in to account to obtain accurate calibration data. 

**Figure 8:**
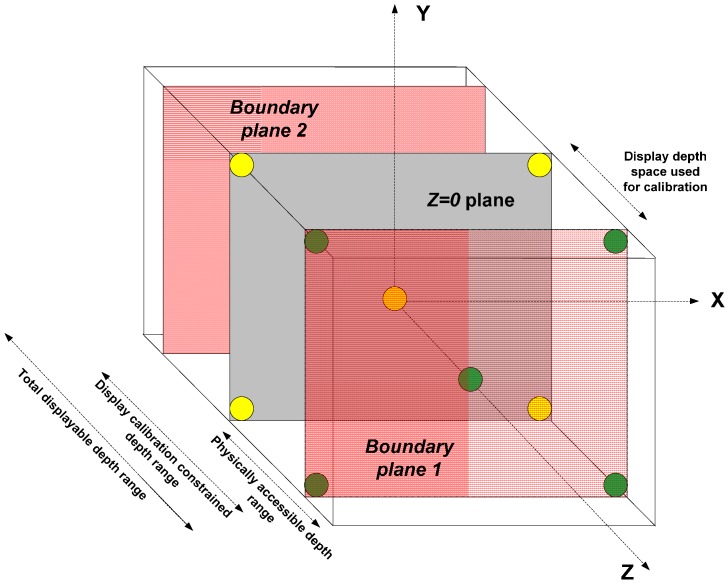
Light field display and Leap Motion Controller calibration: Depth volume bounded by the screen plane and physically accessible constrained boundary plane is calibrated to a comparable sized volume of Leap Motion Controller. Yellow circles show the markers drawn on the screen plane and green circles show markers drawn on boundary plane 1 in the figure.

To minimize the error resulting from the non-uniform spatial resolution and calibration accuracy, we limit the total available depth range of the display for this experiment. We define two boundary planes within the total displayable depth range where the apparent spatial resolution and calibration accuracy is almost the same as on the screen (see Figure 8). This is done by measuring the size of a single pixel at various depths and comparing it with the size of the pixel on the screen plane (for information on pixel size calculation, please refer to [27]). Markers are drawn on the surfaces of the screen plane and on the physically accessible boundary plane and their positions in the display space and Leap Motion Controller space are recorded simultaneously. The calibration should produce a transform ***Ω*** between the two coordinate systems that minimizes the sum of Euclidean distances between the original and projected points when the set of all 3D points in one system is transformed to another. 

Let the $P_{i}^{disp}\in {\mathbb{R}}^{3}$ be the position of *i*th voxel in the display coordinate system, let the $P_{i}^{leap}\in {\mathbb{R}}^{3}\;$be the position of *i*th voxel in the Leap Motion Controller coordinate system and let the $P_{i}^{projleap}\in {\mathbb{R}}^{3}$ be the position of *i*th voxel in the Leap Motion Controller space projected into the display space, where *i* is the index of the current voxel. Then $P_{i}^{projleap}$ and $P_{i}^{leap}$ are related as following:

(2) $$P_{i}^{projleap}=\Omega *\;P_{i}^{leap}$$

where$\;\;\Omega \in {\mathbb{R}}^{4X4}$ is the required transform between two coordinates system. Thus, ***Ω*** should minimize

(3) $$\overset{n-1}{\underset{i=0}{\sum }}{\left(\mu_{i}*Euclidean\;dist\left(P_{i}^{disp},P_{i}^{projleap}\right)\right)}^{\;}$$

where *n* is the number of discrete voxels within the comfortable viewing range outside the display and the constant *µ_i_* is given by the Equation (4).

(4) $$\mu_{i}=\{\begin{array}{c} 1,\\ 0, \end{array}\begin{array}{c} if\;i^{th}\;display\;voxel\;is\;used\;for\;calibration\\ \;\;\;\;\;\;\;if\;i^{th}\;display\;voxel\;is\;not\;used\;for\;calibration \end{array}$$

Thus using homogeneous coordinates, any coordinate $\left(\begin{array}{ccc} x_{\;}^{leap}, & y_{\;}^{leap}, & z_{\;}^{leap} \end{array}\right)$ in the Leap Motion Controller space can be transformed (based on Equation (2)) to the display spaces coordinates $\left(\begin{array}{ccc} x_{\;}^{disp}, & y_{\;}^{disp}, & z_{\;}^{disp} \end{array}\right)$:

(5) $$\left(\begin{array}{c} x_{\;}^{disp}\\ \begin{array}{c} y_{\;}^{disp}\\ z_{\;}^{disp} \end{array}\\ 1 \end{array}\right)=\Omega^{4X4}\left(\begin{array}{c} x_{\;}^{leap}\\ \begin{array}{c} y_{\;}^{leap}\\ z_{\;}^{leap} \end{array}\\ 1 \end{array}\right)$$

Substituting (2) in (3), the required affine transformation matrix should minimize the following energy function:

(6a) $$\overset{n-1}{\underset{i=0}{\sum }}\left(\mu_{i}*Euclidean\;dist\left(P_{i}^{disp},\Omega P_{i}^{leap}\right)\right)$$

(6b) $$\mathrm{subject}\;\mathrm{to}\;P_{j}^{disp}=\Omega P_{j}^{leap},\;j=0,1,2,3\ldots .,m-1$$

where *m* is the number of markers used for calibration. 

We use OpenCV libraries [28] to solve the above optimization problem, which also eliminates any possible outliers in the calibration process. As both the display and the Leap Motion Controller volumes are finite and bounded, it is enough to render markers along the corners of interaction volume bounding box. Although eight corner markers are enough for acquiring a good calibration (total error less than 1 μm), we observed that using ten markers improves the accuracy even further. The additional two markers are placed at the centroids of the two z-bounding planes in display space (see Figure 8). Increasing the number of markers beyond ten has no considerable effect on calibration accuracy. In our case, the spatial resolution of the display changes with depth, according to the equation:

(7) $$s\left(z\right)=s_{0}+2∥z∥\tan\left(\frac{\Phi }{2}\right)$$

where $s_{0}$ is the size of the pixel on the screen, *z* is the distance between current voxel and surface of the screen and φ is the aperture angle of display light rays. During interaction, to account for the varying depth resolution within the defined boundary planes, we formulate a Sphere of Confusion (SoC) and allow the registration of user’s finger position anywhere within the sphere centered at the current 3D position. The radius of SoC is a function of depth from the surface of the screen.

**Figure 9:**
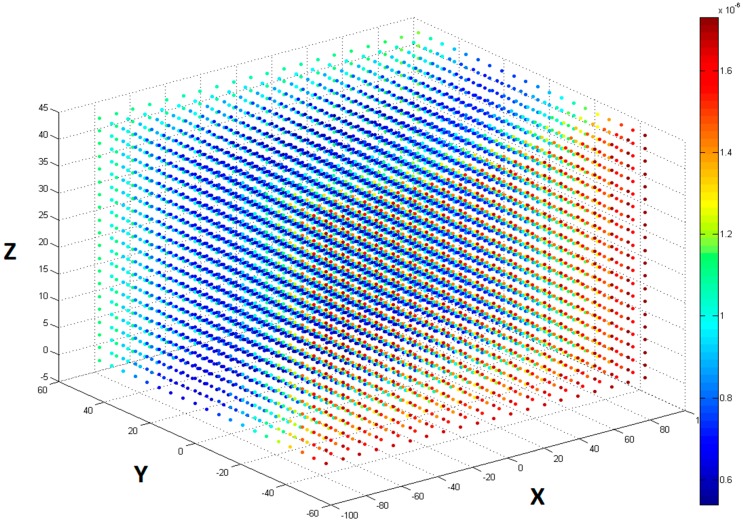
Calibration errors on a uniformly sampled grid in Leap Motion Controller space after projecting to display space.

In order to quantify the calibration results accuracy, we sample the Leap Motion Controller space uniformly with 20 samples along each dimension (8000 samples in total). The distance between adjacent samples is 9 mm in the horizontal direction and 5 mm in the vertical direction. We project these samples individually on to the display space using the calculated transform ***Ω*** and record the Euclidean distance between the original and projected point. Figure 9 shows the projection errors made at various positions on a uniform grid by a sample calibration process. As we can see from the figure, the calibration error is less than 1 μm in most of the places. This is negligible compared to human finger tremor (the order of magnitude of a millimeter) or even Controller’s accuracy.

## 4. Evaluation

### 4.1. Design

**Figure 10:**
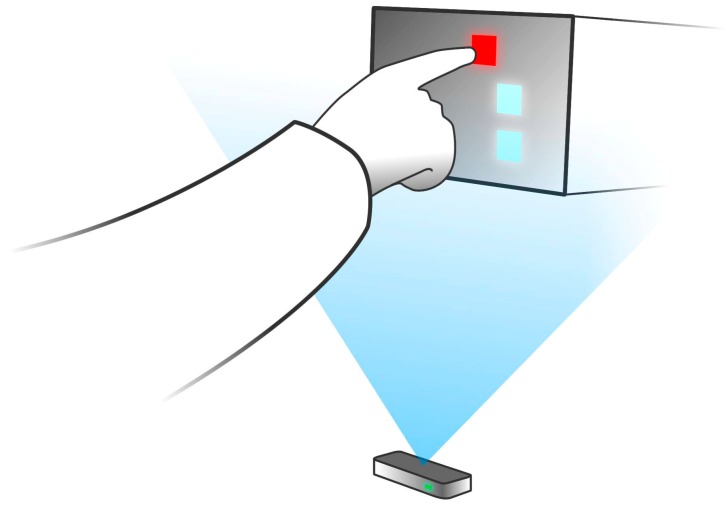
Interaction with the light field display using Leap Motion Controller as finger tracking device.

The proposed freehand interaction with the light field display was evaluated through a simple within-subject user study with 12 participants. Three tiles of the same size were displayed simultaneously and the participants were asked to point (touch) the surface of the red tile as perceived in space (Figure 10). The positions of the tiles varied from trial to trial to cover the entire FOV of the display.

3D and 2D display modes were used representing two different experimental conditions:

- in 2D mode, the displayed objects were distributed on a plane in close proximity of the display surface; and
- in 3D mode, the objects were distributed in a space with the distance varying from 0 to 7 cm from the display.

The 2D mode provided a control environment, which was used to evaluate the specifics of this particular interaction design: the performance and properties of the input device, display dimensions, specific interaction scenario (e.g., touching the objects), *etc*.

Each participant was asked to perform 11 trials within each of the two conditions. The sequence of the conditions was randomized across the participants to eliminate the learning effect.

The light field display and the interaction design were evaluated from the following aspects:

- task completion times,
- cognitive workload, and
- perceived user experience.

The task completion time was measured from the moment when a set of tiles appeared on the display until the moment the user touched the red tile (e.g., hovered over the area where the red tile was displayed within a specific spatial margin of error (15 mm) and for a specific amount of time (0.5 s)).

The cognitive workload was measured through the NASA TLX (Task Load Index) questionnaire, which provides a standardized multi-dimensional scale designed to obtain subjective workload estimates [29]. The procedure derives an overall workload score on the basis of a weighted average of ratings on the following six subscales: “Mental Demands”, “Physical Demands”, “Temporal Demands”, “Own Performance”, “Effort” and “Frustration”.

The perceived user experience was measured through UEQ (User Experience Questionnaire) [30]. which is intended to be a user-driven assessment of software quality and usability. It consists of 26 bipolar items, each to be rated on a seven-point Likert scale (1 to 7). The UEQ algorithm derives a quantified experience rated using the six subscales labeled “Attractiveness”, “Perspicuity”, “Efficiency”, “Dependability”, “Stimulation” and “Novelty” of the technology evaluated.

### 4.2. Results

**Figure 11:**
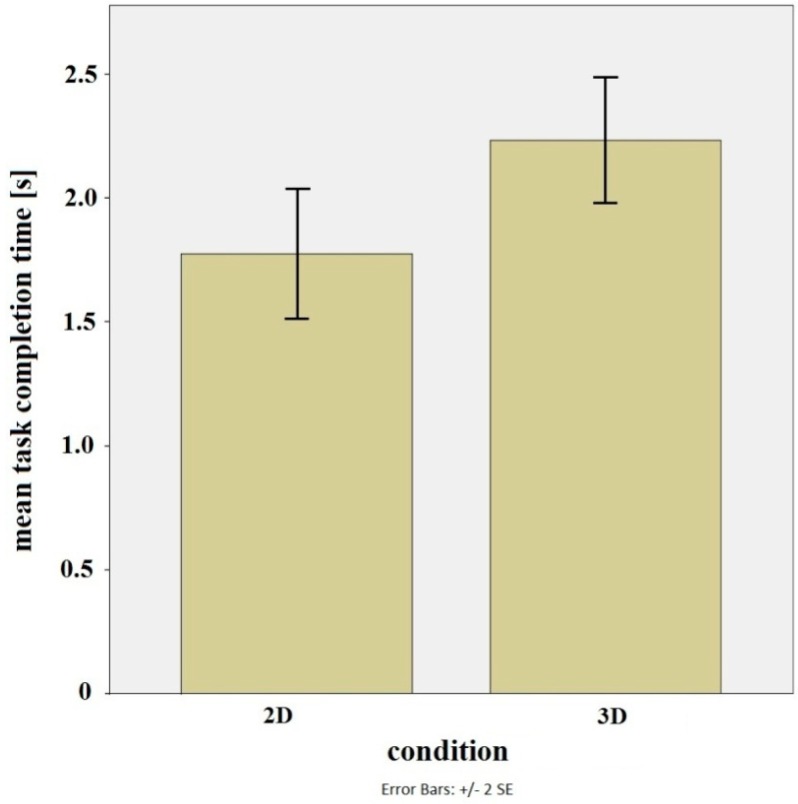
Mean task completion times for the interaction with the objects in 2D and 3D.

Figure 11 shows mean task completion times for both conditions. The results of the T-test showed the interaction in 3D to be significantly slower than the interaction in 2D (t(22) = 2.521, p = 0.019). This result was expected since the additional dimension implies extra time that is needed to, firstly, cognitively process the visual information and, secondly, to physically locate the object in space.

**Figure 12:**
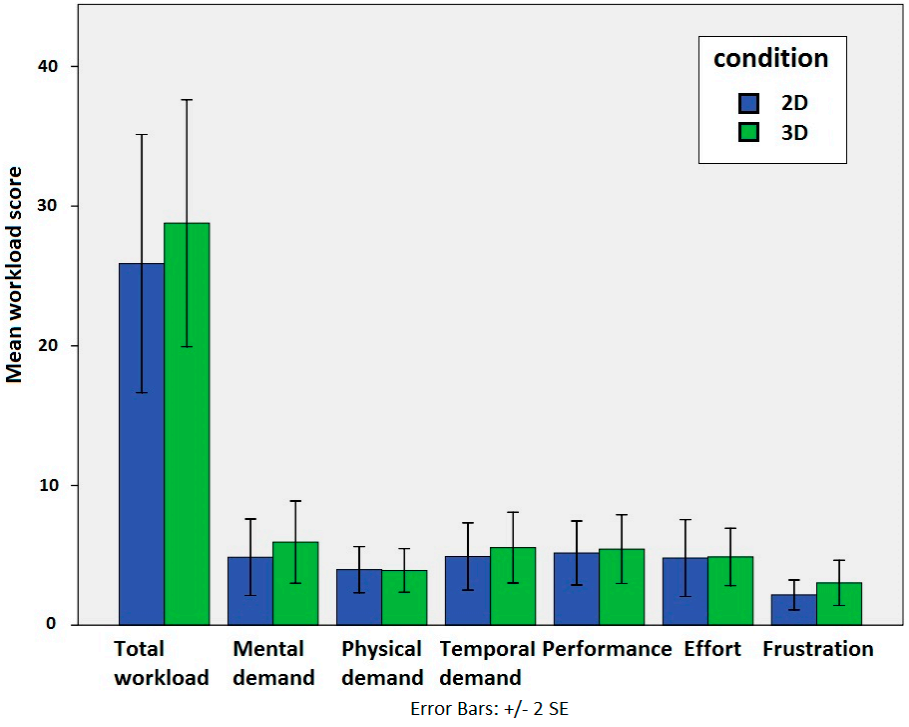
Total workload score and workload scores on the individual subscales of the NASA TLX (Task Load Index) test.

Figure 12 shows mean workload scores for the subscales of the NASA TLX test as well as the overall workload score. The results of the T-test (t(22) = −0.452, p = 0.655) reveal no significant difference in cognitive workload between the conditions.

Similarly, the results of the UEQ also did not reveal any significant differences between both conditions in overall user experience score as well as in the majority of the individual subscales of the test. In other words, the users did not perceive any significant differences between the conditions in terms of general impression, the easiness to learn how to interact with the content, the efficiency of such interaction, the reliability or the predictability of the interfaces used and the excitement or the motivation for such an interaction. The exception is the “novelty” subscale where a tendency towards higher preferences for the 3D mode can be observed.

The analysis of the post-study questionnaire revealed that the rendered objects were seen clearly in both experimental conditions. However, the users favored the 3D mode in terms of rendering realism. When asked to choose the easiest mode, the users’ choices were equally distributed between both modes. However, when asked which mode led to more mistakes in locating the exact object position, two-thirds indicated the 3D mode, which is reflected also in longer task completion times in this particular mode. Finally, when asked about their preference, two-thirds of the participants chose the 3D mode as their favorite one. 

## 5. Discussion and Conclusions

Light field displays allow more realistic and accurate rendering of 3D objects compared to other 3D rendering technologies and have therefore the potential to provide more intuitive and natural interaction in a variety of environments and user scenarios. In this paper, we explained basic functioning of light field displays including the most important advantages and limitations. We proposed a design of a direct touch freehand interaction with a light field display, which included touching (selecting) the objects in at different depths in a 3D scene. The relatively accurate and affordable Leap Motion Controller was used as an input device providing desktop-based user-tracking device.

One of the issues addressed in this paper is a calibration procedure providing the transformation of 3D points from the Controller’s to the display’s coordinate system to get the uniform definition of position within the interaction space. This transformation is of vital importance for accurate tracking of users’ gestures in the displayed 3D scene enabling interaction with the content and manipulation of virtual objects in real-time. The available interaction space has to be selected and limited based on the limitations of the Controller’s effective sensory space as well as the display’s non-uniform spatial resolution. The proposed calibration process results in an error less than 1 μm in a large part of interaction space.

The proposed interaction setup was evaluated by comparing the 3D interaction (e.g., pointing and touching) with objects in space to the traditional 2D touch of objects in a plane. The results of the evaluation revealed that more time is needed to select the object in 3D than in 2D. This was expected, since the additional dimension undoubtedly implies extra time that is needed to, firstly, cognitively process the visual information and, secondly, to physically locate the object in space. However, the poor performance of the interaction in 3D may also be contributed to by the intangibility of the 3D objects and the lack of tactile feedback. This is also in accordance with the findings of other similar studies (e.g., [5]) where poor performance in determining the depth of the targets was related to the intangibility of the objects.

Another, perhaps even more interesting finding was a relatively low cognitive demand of interaction in 3D environment, which was comparable to the simplified 2D interaction scenario. This reflects the efficiency and the intuitiveness of the proposed interaction setup and the freehand interaction with 3D content in general. In the 2D environment, the touch screens built in the majority of smart phones and other portable devices also represented such intuitive and simple input devices enabling the adoption of these technologies by users of all ages and different computer skills. They successfully replaced computer mice and other pointing devices (e.g., very effective and widely used in a desktop environment) and their main advantage was the introduction of the “point and select” paradigm, which seems to be very natural and intuitive. We believe the proposed freehand interaction setup could represent the next step in this transition enabling such direct selection and manipulation of content also in 3D environment. This assumption was confirmed also by the high preference of the users for the proposed setup expressed in the UEQ questioners

The Leap Motion Controller sometimes produced anomaly readings, such as reporting identical position although the finger had moved, reporting false positions far away from the actual finger position or suddenly dropping out of recognition. These anomalies, however, were usually short-termed and did not represent a significant impact on the user’s performance and the overall results. Nevertheless, as such anomalies were known to happen [16] and therefore expected, the study was designed to cope with them: the conditions were randomized and a large number of trials was used within each condition so the anomalies were uniformly distributed among both conditions.

In our study we observed and evaluated only the process of selection of an object in a 3D scene. In general, more sophisticated actions can be performed while interacting with 3D content, such as advanced manipulation (e.g., changing position and orientation of virtual objects), changing of viewpoint of the scene, *etc*. In the future we are planning on evaluating the proposed interaction setup in a game-like scenario including both hands. Users will be asked to move different objects within the 3D scene and change their orientation or even change their shape. 

Another aspect that needs to be evaluated in the future is the interaction with tactile (or/and some other kind of) feedback so the interaction will be similar to touch-sensitive surfaces in 2D. Finally, the comparison in performance between Leap Motion Controller and other, especially classic and user-familiar input devices, like computer mouse, will be interesting.
